# Familial dilated cardiomyopathy with RBM20 mutation in an Indian patient: a case report

**DOI:** 10.1186/s43044-021-00165-6

**Published:** 2021-05-22

**Authors:** Soumi Das, Sandeep Seth

**Affiliations:** Department of Cardiology, All India Institute of Medical Science, New Delhi, India

**Keywords:** Dilated cardiomyopathy, RBM20, Autosomal, Sudden cardiac death, Case report

## Abstract

**Background:**

Dilated cardiomyopathy (DCM) is a disease of the heart muscle characterized by ventricular dilation and a left ventricular ejection fraction of less than 40%. Unlike hypertrophic cardiomyopathy (HCM) and arrhythmogenic right ventricular cardiomyopathy (ARVC), DCM-causing mutations are present in a large number of genes. In the present study, we report a case of the early age of onset of DCM associated with a pathogenic variant in the RBM20 gene in a patient from India.

**Case presentation:**

A 19-year-old Indian male diagnosed with DCM was suggested for heart transplantation. His ECG showed LBBB and echocardiography showed an ejection fraction of 14%. He had a sudden cardiac death. A detailed family history revealed it to be a case of familial DCM. Genetic screening identified the c.1900C>T variant in the RBM20 gene which led to a missense variant of amino acid 634 (p.Arg634Trp).

**Conclusion:**

To the best of our knowledge, the variant p.Arg634Trp has been earlier reported in the Western population, but this is the first case of p.Arg634Trp in an Indian patient. The variant has been reported to be pathogenic at an early age of onset; therefore, close clinical follow-up should be done for the family members caring for the variant.

## Background

Dilated cardiomyopathy (DCM) is characterized by ventricular dilation, impaired systolic function, reduced myocardial contractility, and a left ventricular ejection fraction of less than 40% with a frequency of 1:250 or greater [1]. Most of the DCM cases are sporadic, but approximately 30–48% have a positive family history [2] with an autosomal pattern of inheritance. Although more than 60 genes are linked with DCM [3], RBM 20 is associated with familial DCM and leads to the early age of onset and high mortality [4]. In the present study, we report heterozygous variant c.1900C>T in a severe case of DCM from India. This is the first case from India with c.1900C>T leading to sudden cardiac death.

## Case presentation

**Fig. 1:**
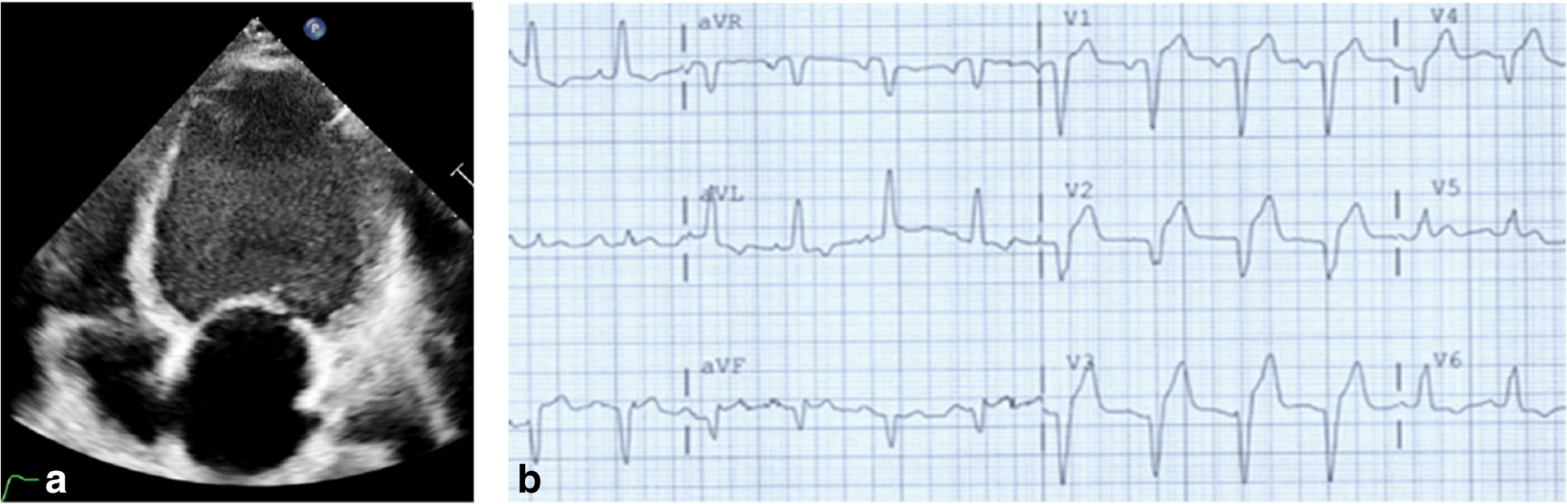
**a** Echocardiogram showing dilated left atrium and left ventricle with poor function. **b** Electrocardiogram showing incomplete LBBB, poor R wave progression

**Fig. 2:**
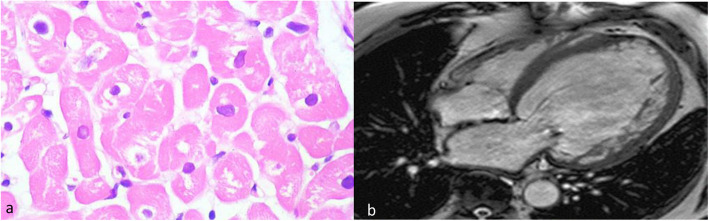
**a** Endomyocardial biopsy of the proband showing hypertrophy, variable nuclear enlargement with sarcoplasmic vacuolar degeneration of the myocytes, and absence of inflammation. **b** Cardiac magnetic resonance imaging of the proband with ejection fraction 14% and dilated thinning out of the left ventricle

A 19-year-old male was diagnosed with dilated cardiomyopathy. After 12 months, the patient was referred to AIIMS New Delhi with shortness of breath and was admitted to AIIMS ICU. His blood pressure was 94/52 mmHg and heart rate 73 beats per minute at the time of admission. NTproBNP, TropT, and CRP levels at the time of admission were 1881 pg/ml, 11.1 pg/ml, and 3 mg/ml, respectively. SGOT and SGPT levels were 76 and 109 units/l, respectively. The patient also had a history of celiac disease. Echocardiographic screening showed severe left ventricular systolic dysfunction with an ejection fraction of 14%, and electrocardiogram showed left bundle branch block (LBBB) (Fig. 1). The patient had no evidence of inflammation which was confirmed by endomyocardial biopsy and cardiac magnetic resonance imaging (Fig. 2). The patient was treated with steroids, IV immunoglobulins, and IV inotropic agents like dopamine and dobutamine at admission and was finally discharged after being stabilized. The patient was then put on diuretics, carvedilol, and sacubitral-valsartan and was advised to undergo a next-generation sequencing which showed heterozygous variant c.1900C>T in the RBM20 gene leading to a missense variant of amino acid 634 (p.Arg634Trp). The patient was readmitted after a month and was listed for heart transplantation but had a sudden cardiac death after 3 months.

**Fig. 3:**
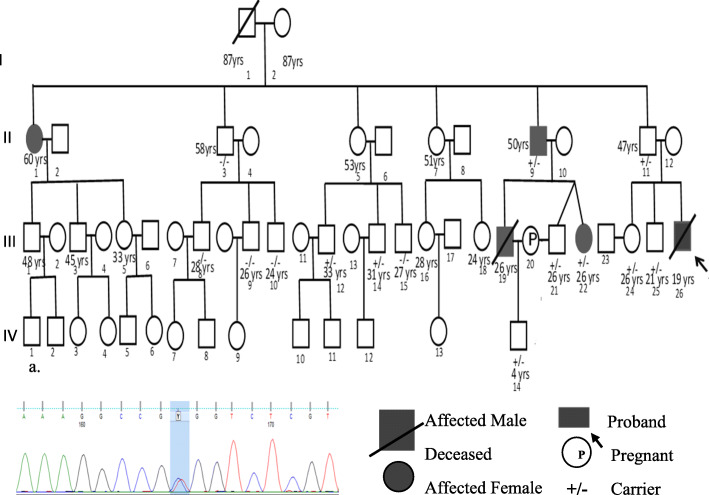
**a** Pedigree of the proband. **b** Electropherogram showing variant c.1900C>T in the RMB20 gene

A detailed family history revealed that one of the cousins (III 19) of the patient diagnosed with DCM had sudden cardiac death at the age of 26 years, and his aunt (II 1) aged 60 years was also affected with DCM (Fig. 3). Further, the family was advised for echocardiography screening and Sanger sequencing for the variant c.1900C>T. Echocardiography screening revealed clinical features of DCM in the deceased cousin’s father aged 50 years (II 9) and his sister aged 26 years (III 22). Both of them were asymptomatic and were put on beta-blockers. Sanger sequencing revealed the patient’s father (II 11), sister (III 24), brother (III 25) uncle (II 9), and cousins (III 12, III 14, III 15, III 21, III 22) were carriers of the variant c.1900C>T. The deceased cousin’s 4-year-old son was also a carrier of the variant (IV 14). The wife of the deceased cousin was married to his brother (II 21) who was expecting a child and was advised to undergo fetal screening for the variant but she refused.

## Discussion

RBM20 is a regulator of heart-specific alternative splicing of Titin (TTN) gene which encodes the largest protein in mammals. Titin protein plays an important role in generating passive tension of cardiomyocytes; thus, regulation of alternative splicing in titin becomes important in normal heart functioning [5]. In an animal model study, it has been reported that deletion of RBM20 leads to the formation of unusually large Titin protein thus leading to DCM [6].

Mutation in RBM20 leads to 2–3% of familial and sporadic dilated cardiomyopathy. Penetrance, age of onset, and severity can vary among patients with the same variants within the family and also between identical twins. RBM 20 leads to arrhythmias, early age of onset, high mortality, and penetrance in most of the cases. In most of the cases, patients with RBM20 undergo heart transplantation or implantation of an ICD [7]. The gene acts in a highly gender-specific manner and affects the males more [7]. RBM20 comprises 14 exons, and the variant c.1900C>T is present in the arginine/serine (RS)-rich region in exon 9. The c.1900C>T variant is highly conserved among species and has been earlier reported by Bruch et al. [4] among white European DCM patients. The variant was absent in 480 control samples [4]. In silico analysis by Mutation Taster, PolyPhen2, SIFT, and FATHMM has predicted the c.1900C>T variant to be damaging [8]. The mechanism by which RBM20 leads to DCM is unclear, but it is predicted that the RS-rich region is involved in protein-protein interaction, and any mutation in this region may affect the ability of RBM20 protein to interact with other spliceosome proteins thus disrupting the normal RNA splicing mechanism [9]. There is a strong physicochemical difference between arginine and tryptophan which is likely to impact secondary protein structure. To the best of our knowledge, this is the first case among the Indian population with the c.1900C>T variant leading to severe heart failure and leading to sudden cardiac death at a very early age. The penetrance of the variant can be observed in all generations, but the severity is high among males. Since the family members remain asymptomatic in most of the cases, therefore, a detailed family history and echocardiographic screening should be done along with genetic screening in case of familial DCM. Those family members who have been carriers of the variant c.1900C>T should undergo an echocardiography screening once annually. Pregnant women in the family are suggested to undergo a fetal screening for the variant.

## Conclusion

We conclude that the RBM20 gene leads to the early age of onset of DCM causing sudden cardiac death and heart transplantation. Therefore, close clinical follow-up should be done in families with RBM20 variants. Male family members with RBM20 variants should undergo echocardiography screening frequently whereas females can be treated in a more conserved way. RBM20 variants lead to arrhythmia; therefore, early ICD implantation and antiarrhythmic drug therapy can be an option for treatment.

## Data Availability

All relevant data supporting the conclusions of this article are included within the article.
